# Supplementary material for: Loss of endogenous tau suppresses APOE4-induced patterned behavioral decline and axon dysmorphia in a model of Alzheimer's disease
Source: Dis Model Mech. 2026 Jul 27;19(7):dmm052469. doi: 10.1242/dmm.052469 (PMC13474567; doi:10.1242/dmm.052469)
Supplement: Supplementary file 1 [file dmm-19-052469-s1.pdf]

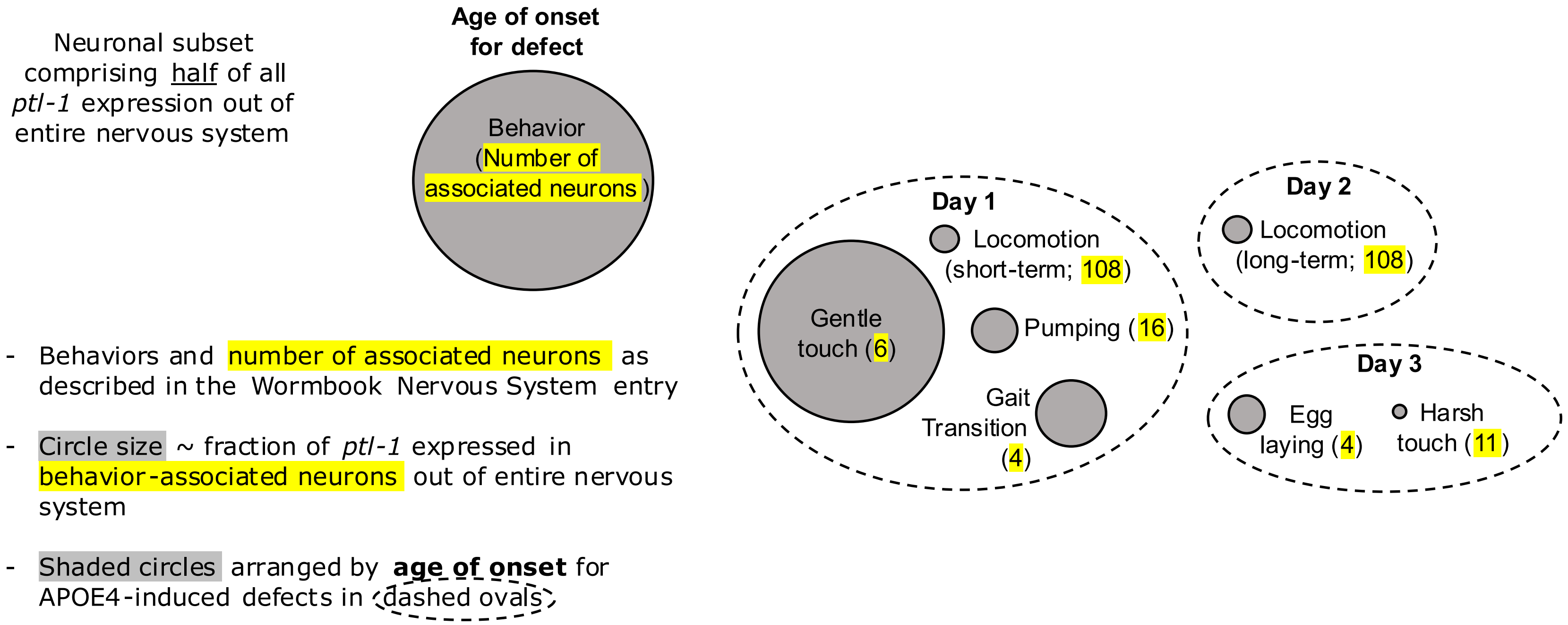

Fig. S1. Behavior-associated neurons vary in number, how much *ptl-1* they express, and timing of potential APOE4-induced dysfunction

Behaviors became defective or abnormal at different ages (grouped by age of onset in dashed ovals). We speculate that this timing may relate to the amount of *ptl-1* expressed across behavior-associated neurons, depicted by the grey circle size nearby each behavior.

Disease Models & Mechanisms • Supplementary information

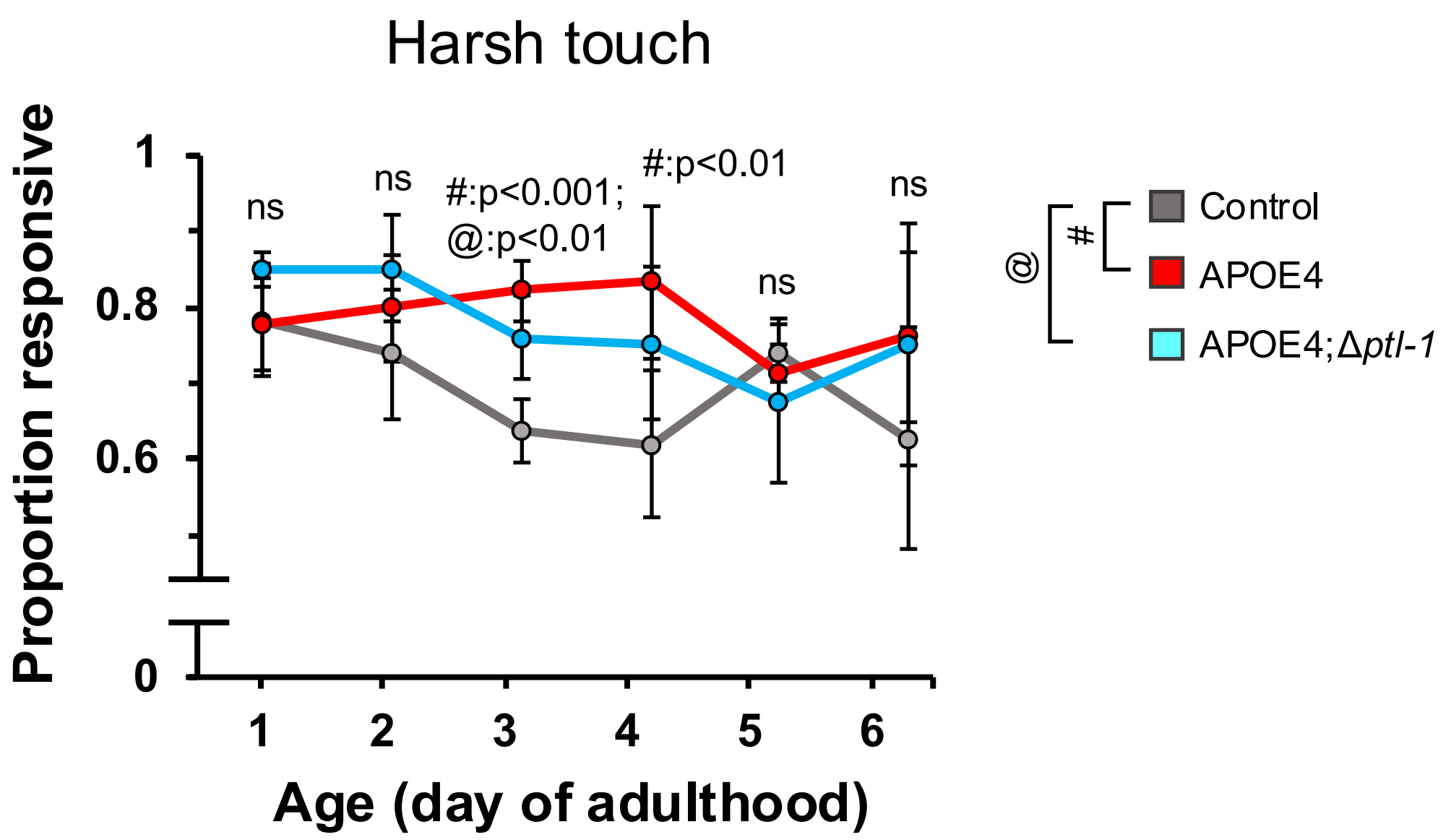

Fig. S2. Continued assessment of harsh touch out to adult day 6 (D6)

Compared to control, APOE4 ($p<0.001$) and APOE4;Δ*ptl-1* ($p<0.01$) animals are more sensitive to harsh touch beginning in D3 adults. N=25 for control, N=26 for APOE4, and N=31 for APOE4;Δ*ptl-1*. To explore this unexpected increase in sensitivity, animals were also tested up to D6 age adults. Compared to D4 control, the increased sensitivity of APOE4 animals remains ($p<0.01$; N=27 for control and N=20 for APOE4), but no statistically significant difference was observed at D5 (N=20 for control and N=20 for APOE4) into D6 (N=12 for control and N=20 for APOE4). All data are represented as mean ± SEM. All statistical comparisons were made using planned tests after two-way ANOVA, where # or @ indicate significant differences between control and APOE4 or control and APOE4;Δ*ptl-1* groups, respectively. N is the total sample size representing all animals across replicates.

Disease Models & Mechanisms • Supplementary information

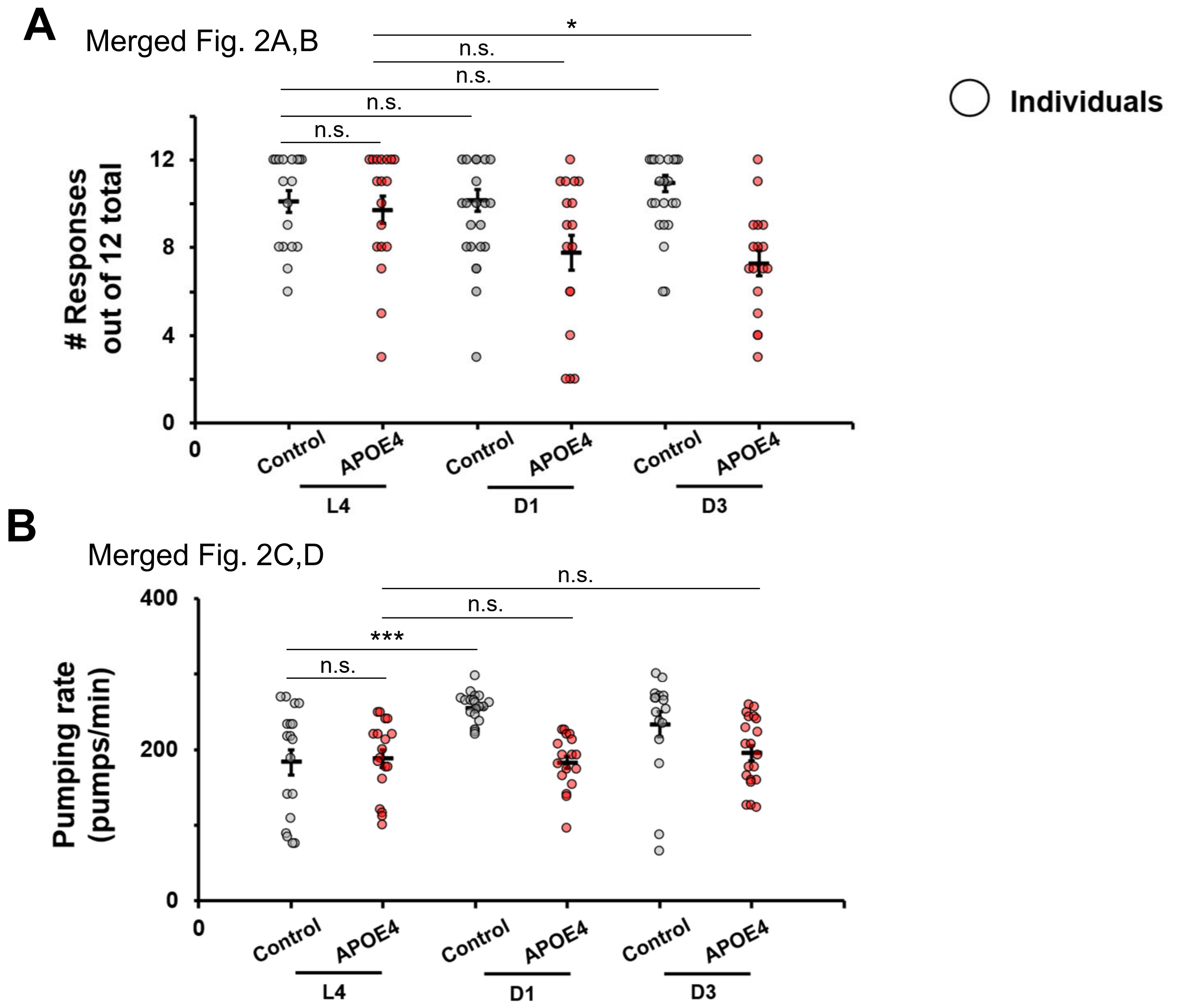

Fig. S3. Replotted: APOE4-induced behavioral decline may be the result of developmental and age-related defects

Replotting of Figure 2A-C to compare strains and days in one graph. (A) Assays of gentle touch sensitivity. (B) Assays of pumping.

Disease Models & Mechanisms • Supplementary information

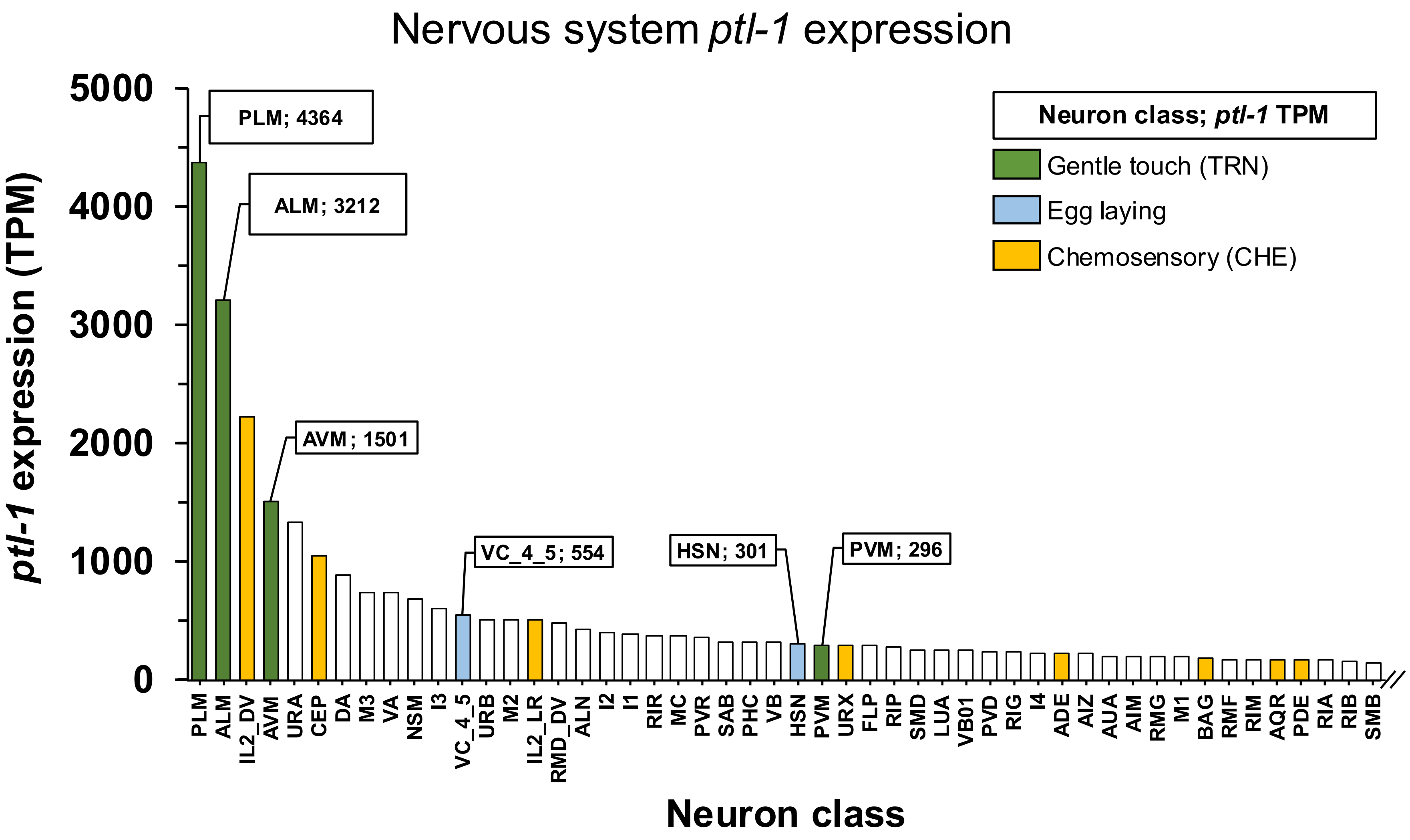

Nervous system ptl-1 expression
ptl-1 expression (TPM)
5000
4000
3000
2000
1000
0
PLM; 4364
ALM; 3212
AVM; 1501
VC_4_5; 554
HSN; 301
PVM; 296
Neuron class; ptl-1 TPM
Gentle touch (TRN)
Egg laying
Chemosensory (CHE)
PLM ALM IL2_DV AVM URA CEP DA M3 VA NSM I3 VC_4_5 URB M2 IL2_LR RMD_DV ALN I2 I1 RIR MC PVR SAB PHC VB HSN PVM URX FLP RIP SMD LUA VB01 PVD RIG I4 ADE AIZ AUA AIM RMG M1 BAG RMF RIM AQR PDE RIA RIB SMB
Neuron class

Fig. S4. Nervous system *ptl-1* expression

Bar plot showing cell-specific RNA sequencing data acquired from CeNGEN data set (Taylor et al., 2021). Green bars represent the touch receptor neurons (TRNs), blue bars represent egg-laying associated neurons (VCs4,5 and HSN), and yellow bars represent the *che-2*-expressing sensory neurons (CHEs).

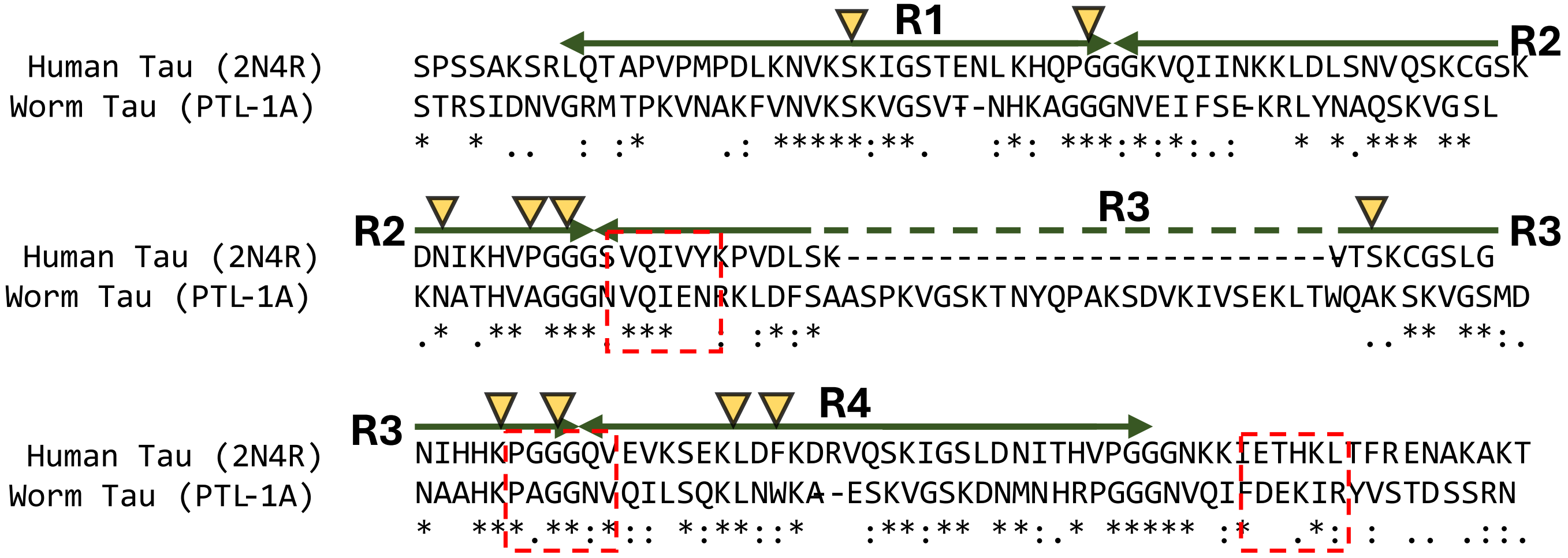

Fig. S5. Disease-relevant tau domains are conserved from human to *C. elegans* Canonical microtubule binding repeats (R1-4) of human tau are marked above the alignment using double-sided arrows. Enclosed by red boxes are protein domains shown to interact with each other within tau aggregates across tauopathies (Reviewed in Oakley et al., 2020). Yellow arrowheads indicate residues whose mutation has been empirically shown to promote or inhibit aggregate formation of human tau (McDermott et al., 1996; Strang et al., 2019); these residues are also conserved in worm PTL-1. Alignment generated with Clustal Omega using publicly available primary sequences for endogenous tau protein in human and worm (The UniProt Consortium; Wormbase). Note that full length tau is not depicted to emphasize the R domains.

Disease Models & Mechanisms • Supplementary information

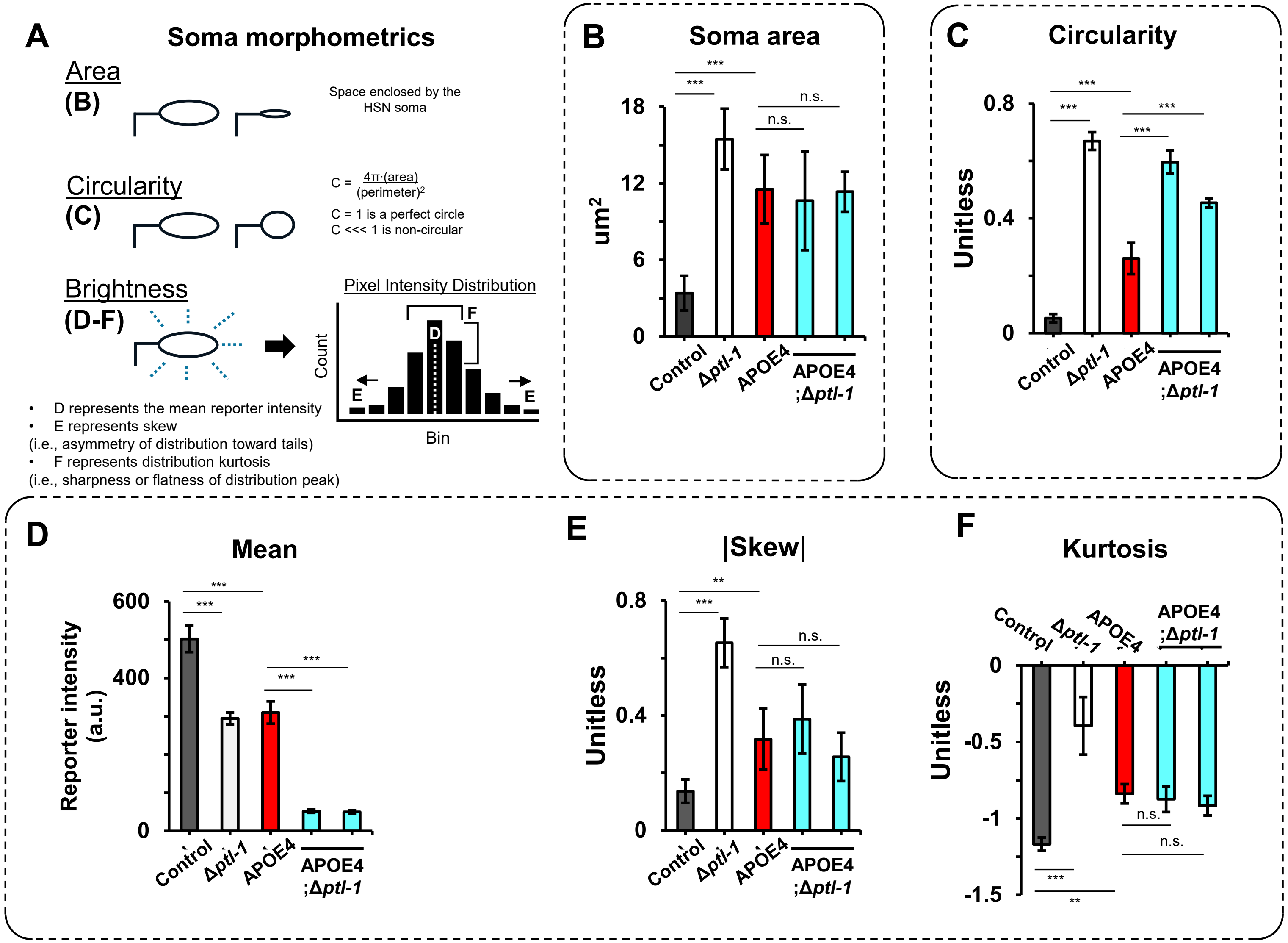

Disease Models & Mechanisms • Supplementary information

Fig. S6. Morphometrics of the HSN soma are inconsistent correlates of APOE4-induced dysfunction

(A) Descriptions of the soma morphometrics quantified in ImageJ with the goal of identifying characteristics which we hypothesized would correlate with our behavioral data (See Fig. 1G).

(B) Quantification of HSN soma area. While APOE4 animals exhibited larger soma on average compared to control, so did APOE4 and APOE4;Δ*ptl-1* groups.

(C) The circularity metric of the HSN soma was not obviously reflective of HSN dysfunction.

(D-F) Quantification of average GFP reporter brightness in the HSN soma.

(D) On average, HSN soma in APOE4 animals were dimmer, qualitatively consistent with our previous observations (Sae-Lee et al., 2020). However, APOE4;Δ*ptl-1* animals exhibit even dimmer HSN soma than APOE4.

(E,F) Quantifications of pixel intensity distribution. APOE4 reporter distributions appear, on average, to be more tail-skewed and exhibit more positive kurtosis compared to control. This could indicate non-uniform filling of GFP in the cytoplasm, but how this reflects HSN health and function is unclear. All data are represented as mean ± SEM. N is the total sample size representing all animals. N=19 for control, N=9 Δ*ptl-1*, N=18 for APOE4, N=12 for APOE4;Δ*ptl-1* (left), and N=12 for APOE4;Δ*ptl-1* (right).

All statistical comparisons were made planned tests after two-way ANOVA. **: $p<0.01$; ***: $p<0.001$.

Disease Models & Mechanisms • Supplementary information

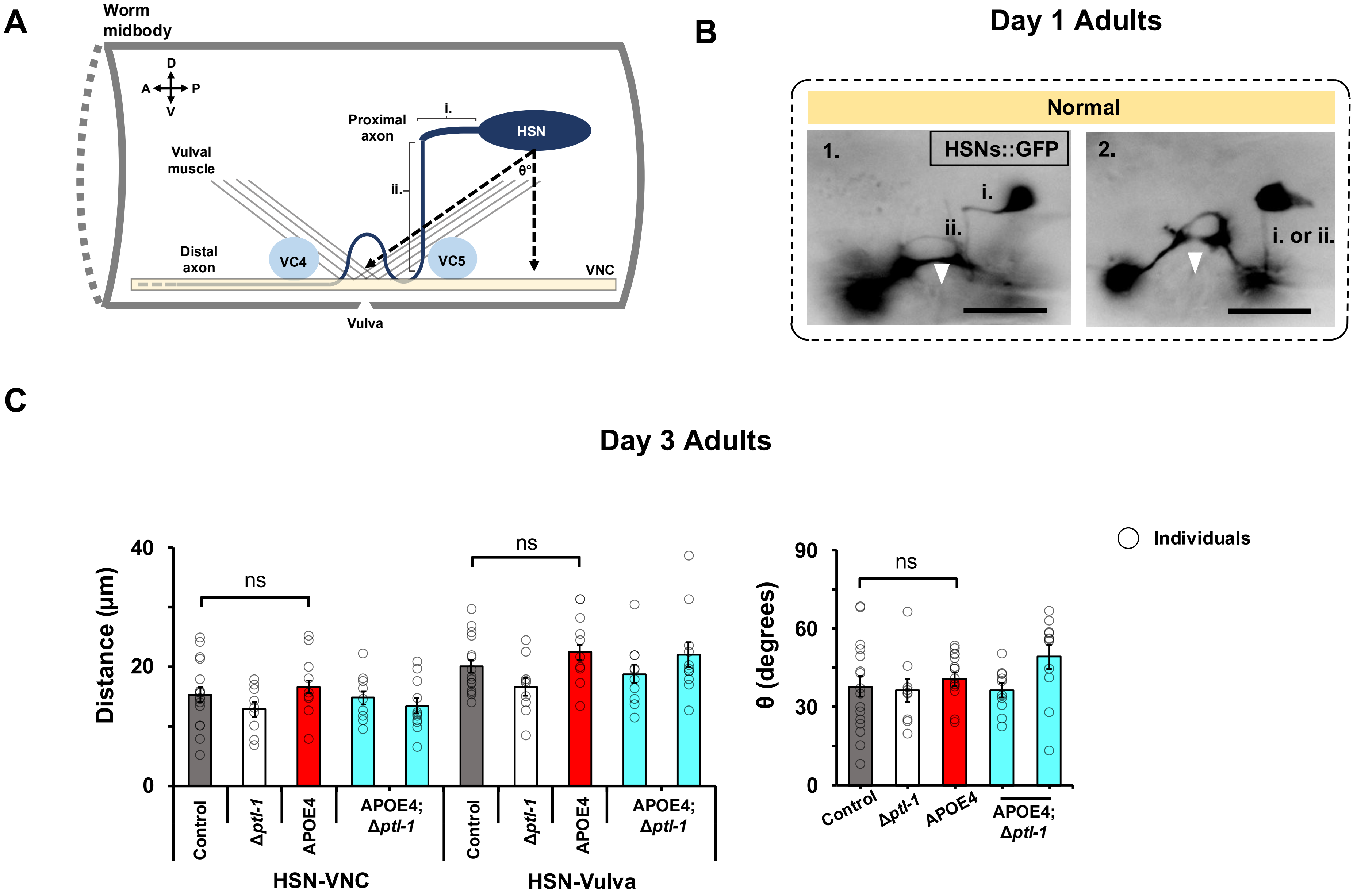

Disease Models & Mechanisms • Supplementary information

Fig. S7. Normal HSN axons in D1 adults and HSN soma positions in D3 adults

(A) Cartoon of the worm midbody, centered about the vulva area, depicting: the HSN proximal axon, the VC4,5 neurons, vulval musculature, and ventral nerve cord (VNC) (B) Representative live images of both normal HSN axon morphologies in D1 adult control animals. The vulva area is also marked with a white arrowhead for anatomical reference. All scale bars are 20 µm. (C) Lengths of vectors measured from either soma to VNC met at a perpendicular or soma to vulva area as indicated by arrowheads of respective dashed lines (left). Consolidated metric of HSN soma position calculated as the angle θ, in degrees, formed between the vectors described above (right). Quantification of HSN soma position shows no significant difference between control and APOE4. N=17 for control, N=9 for Δ*ptl-1*, N=16 for APOE4, N=10 for APOE4;Δ*ptl-1* (left), and N=11 for APOE4;Δ*ptl-1* (right). Statistical comparisons were made using planned comparisons after two-way ANOVAs.

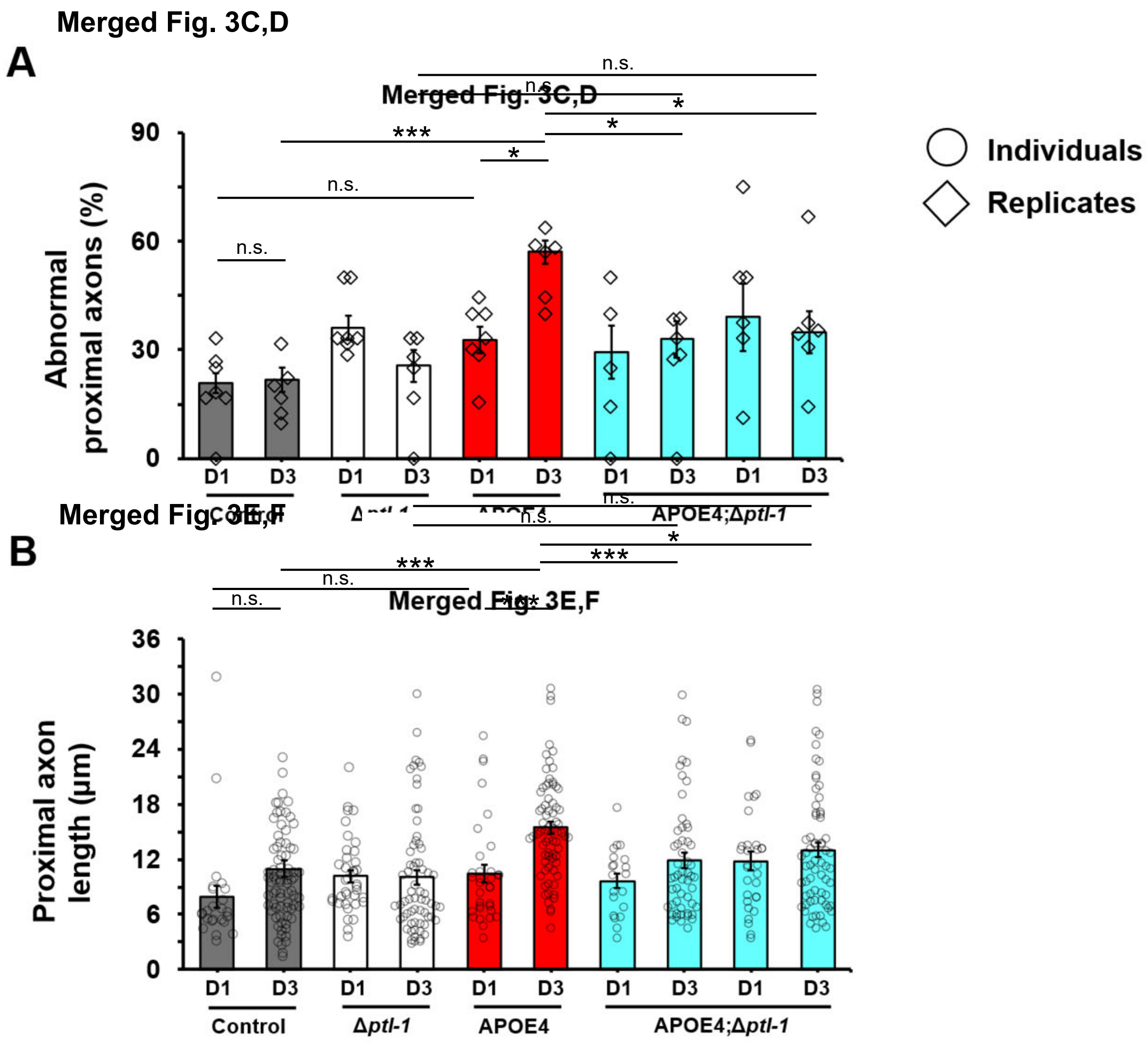

Disease Models & Mechanisms • Supplementary information

Fig. S8. Replotted Figure 3: Morphometrics of the HSN soma are inconsistent correlates of APOE4-induced dysfunction Replotted Figure 3C-F to compare strains and days in one graph. (A) Percent of abnormal HSN axon shape. (B) Length of proximal HSN axon. Statistical comparisons were made using planned pairwise made using χ^2 tests of independent for (A) and comparisons after two-way ANOVAs for (B). *: $p<0.05$; ***: $p<0.001$.

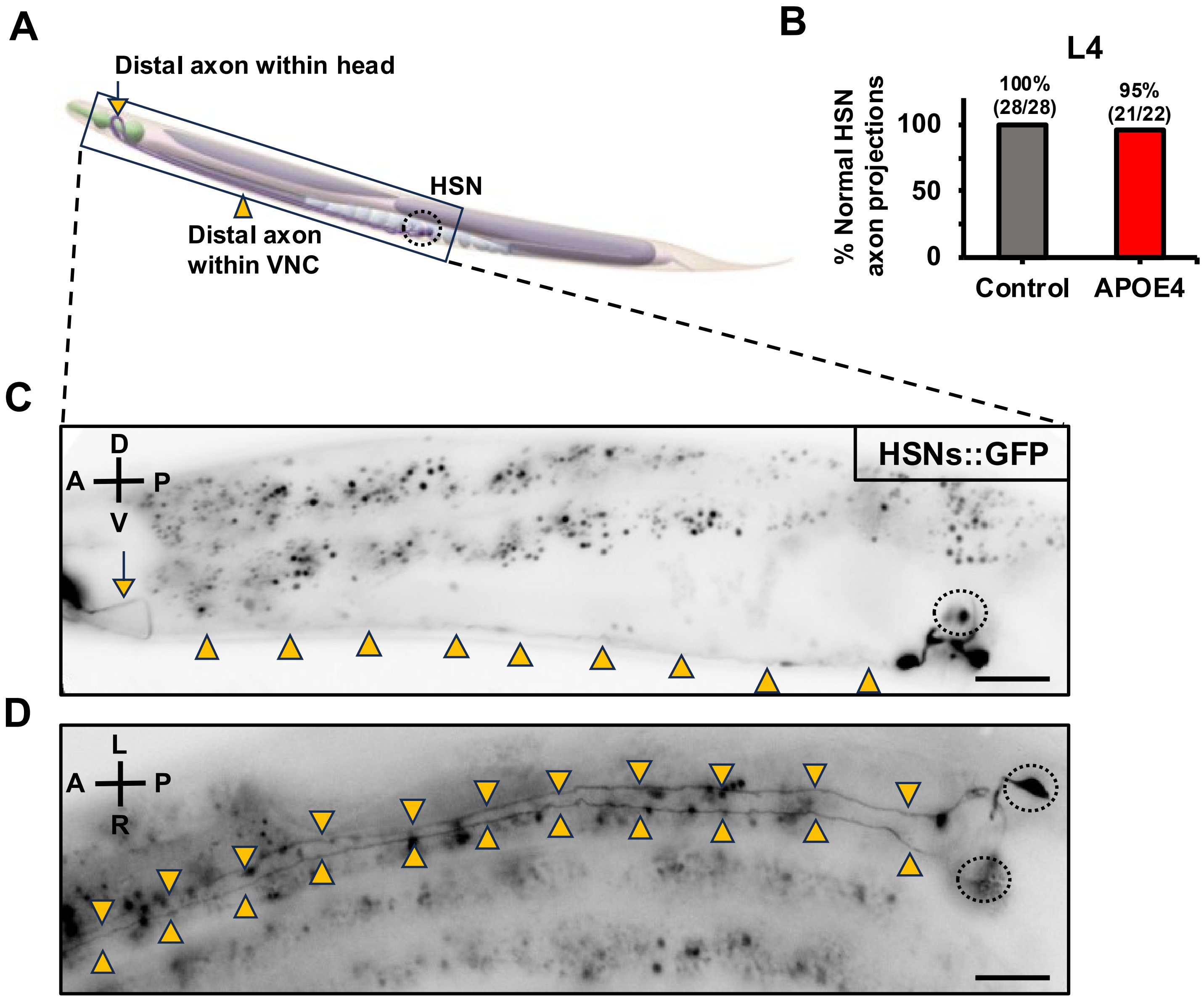

Disease Models & Mechanisms • Supplementary information

Fig. S9. HSN axons projection is normal at L4 developmental stage in APOE4 worms
(A) Cartoon depicting the midbody and head areas of the worm body through which the HSN axon typically projects.
(B) Quantification of animals with normally projecting axons through the ventral nerve cord (VNC) into the head in control (N=28) and APOE4 (N=22) worms.
(C,D) Representative live images of HSN (soma in dashed circle) axon projections in the VNC (arrowheads) and head (arrow) from animals in (C) lateral or (D) ventral orientations. All scale bars are 20 µm.

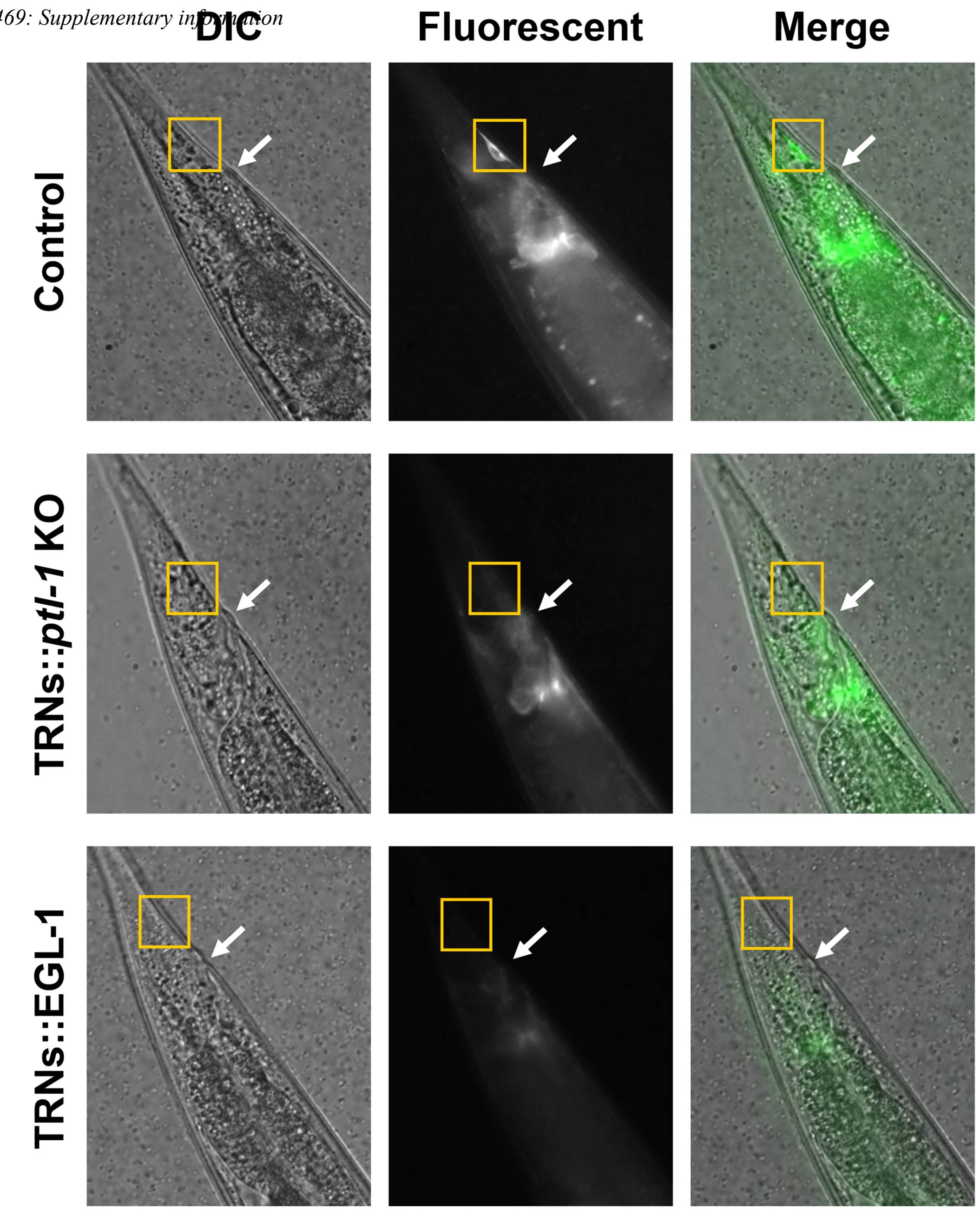

Disease Models & Mechanisms • Supplementary information

Fig. S10. Confirmation of PLM neuron cell body presence or absence using Differential Interference Contrast (DIC) and fluorescent microscopy. Representative DIC and fluorescent micrographs of tail of different strains. (Top) Soma of PLM touch neuron in the tail is visible with DIC optics and mNeonGreen fluorescently tagged PTL-1 in control worm. (Middle) PLM soma is present but the mNG tagged PTL-1 is absent in the TRNs::ptl-1 worm. (Bottom) PLM soma and mNG tagged PTL-1 are both absent in the TRNs::EGL-1 worm in which the TRNs are genetically ablated.

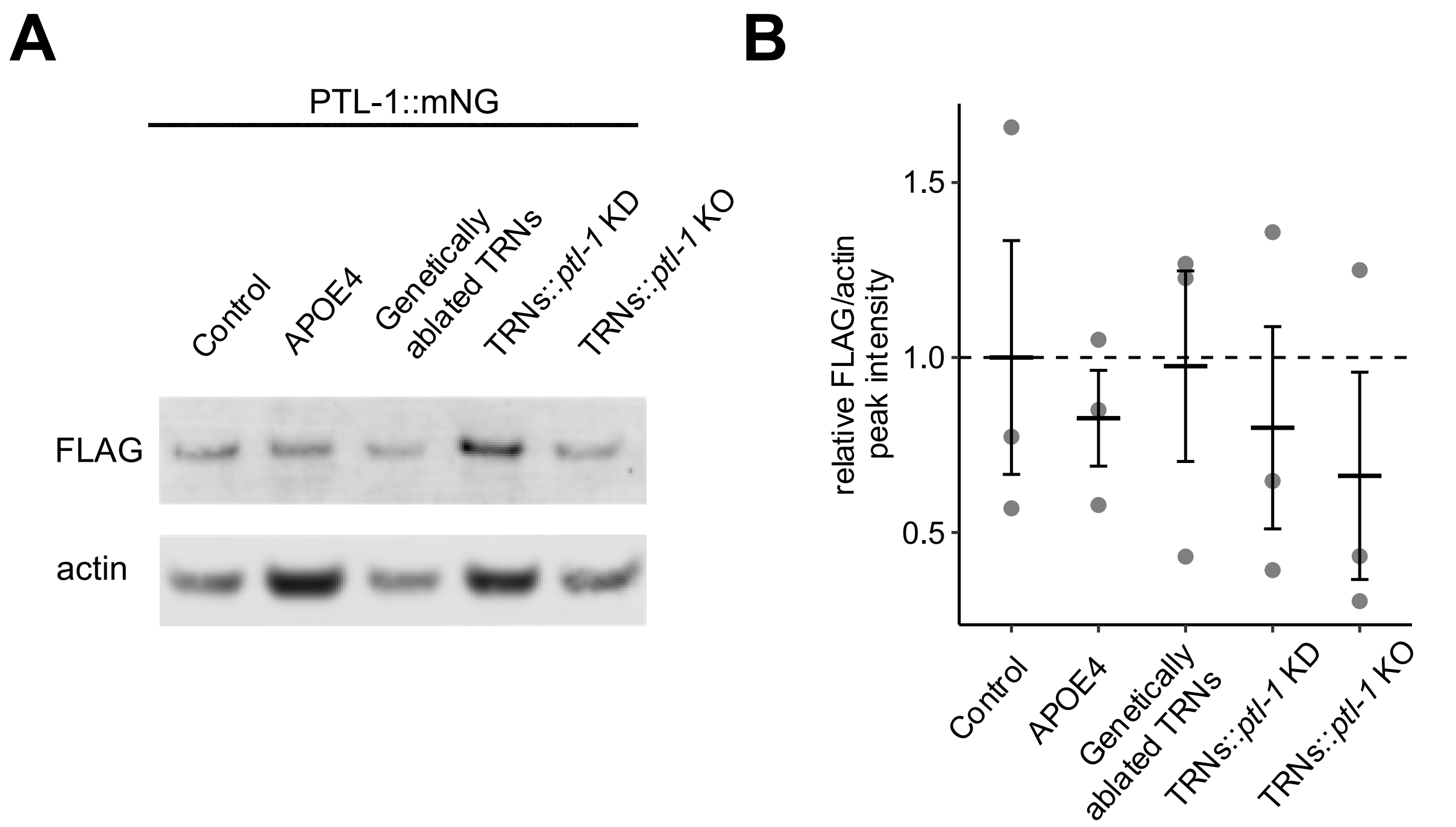

Fig. S11. Whole-animal PTL-1 content from control strains with PTL-1 depleted in the TRNs (left) Representative Western blot to detect PTL-1::mNG. The expected band of ~76kDa was detected by probing for the FLAG epitope engineered into this tag (Krieg et al., 2017). (right) Western blot quantification of FLAG bands normalized to actin bands in *ptl-1*::*mNG* strains revealed variably levels of intensity for control (strain GN655) and the TRNs::EGL-1 (Genetically ablated TRNs), TRNs::*ptl-1* KD, TRNs::*ptl-1* KO strains used in Figs. 4 and 5.

Disease Models & Mechanisms • Supplementary information

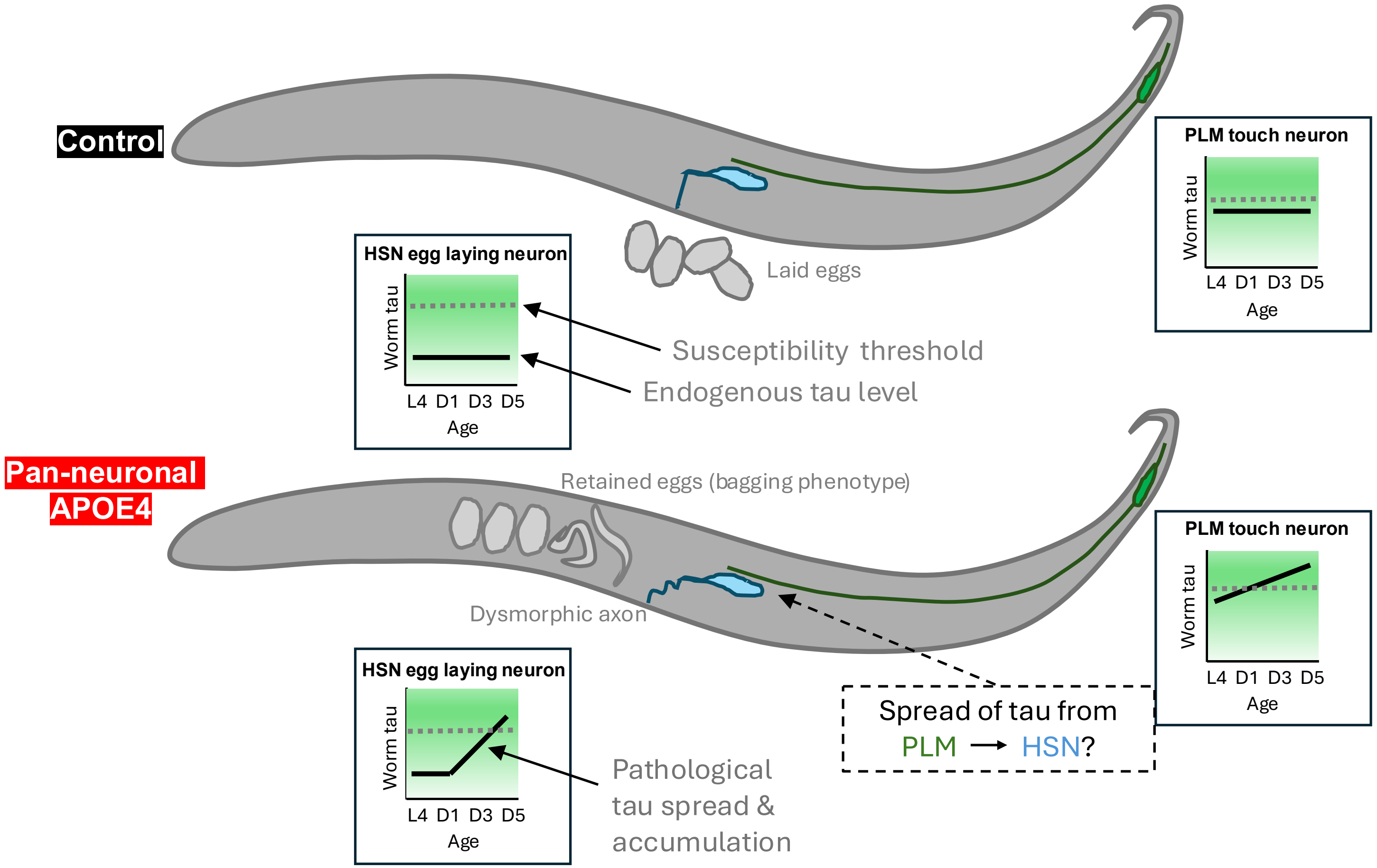

Control
PLM touch neuron
Worm tau
L4 D1 D3 D5
Age
Laid eggs
HSN egg laying neuron
Worm tau
L4 D1 D3 D5
Age
Susceptibility threshold
Endogenous tau level
Pan-neuronal
APOE4
Retained eggs (bagging phenotype)
Dysmorphic axon
PLM touch neuron
Worm tau
L4 D1 D3 D5
Age
HSN egg laying neuron
Worm tau
L4 D1 D3 D5
Age
Pathological
tau spread &
accumulation
Spread of tau from
PLM → HSN?

Fig. S12. Working model: Neuronal dysfunction and dysmorphia relate to different intrinsic levels and interneuron spread of worm tau. In the control worm, HSN and PLM neurons function and appear normal when their levels of worm tau (PTL-1) are below a susceptibility threshold. In the APOE4 worm, APOE4 induces PTL-1 levels to rise above a pathological susceptibility threshold due to 1) accumulation within a neuron, and/or 2) synaptic spread. In this model, the PLM neuron becomes dysfunctional before the HSN neuron because its intrinsic levels of PTL-1 are higher. HSN neuron becomes dysfunctional and dysmorphic next because of pathological spread and accumulation.
